# Smoking may be a risk factor for carpal tunnel syndrome: Insights from Mendelian randomization analysis

**DOI:** 10.18332/tid/199930

**Published:** 2025-01-30

**Authors:** Wei Shi, Kaixuan Wu, Hui Li, Huafeng Zhang

**Affiliations:** 1 Department of Orthopedics, Tianjin Medical University General Hospital, Tianjin, People’s Republic of China; 2 Department of Orthopedics, Tianjin University Central Hospital, Tianjin, People’s Republic of China

**Keywords:** carpal tunnel syndrome, GWAS, pain, risk factor, smoking

## Abstract

**INTRODUCTION:**

It is currently uncertain whether smoking is a risk factor for carpal tunnel syndrome (CTS). This study aims to elucidate association between smoking and CTS using Mendelian randomization (MR) analysis.

**METHODS:**

This study was a secondary analysis of publicly available GWAS data, using four smoking phenotypes (smoking initiation, smoking status, lifetime smoking, and never smoking) as exposures, and two CTS datasets (discovery and validation sets) as outcomes for MR analysis. The discovery set (n=480201) was used to explore the causal relationship between smoking and CTS, while the validation set (n=385304) was used to confirm the results. The effects of smoking on CTS were assessed using inverse variance weighted (IVW), MR-Egger, and weighted median methods. Cochran’s Q test was used to detect heterogeneity, and MREgger to test for pleiotropy. Finally, a meta-analysis was performed on the IVW results from both the discovery and validation sets.

**RESULTS:**

IVW results showed that in both the discovery and validation sets, smoking initiation, smoking status, and lifetime smoking are risk factors for CTS. The summary results from the meta-analysis are as follows: smoking initiation (OR=1.17; 95% CI: 1.08–1.27, p<0.001), smoking status (OR=1.87; 95% CI: 1.56–2.24, p<0.001), and lifetime smoking (OR=2.46; 95% CI: 2.03–3.00, p<0.001). Conversely, never smoking is a protective factor against CTS, with the summary result of the meta-analysis being: OR=0.55; 95% CI: 0.42–0.71, p<0.001.

**CONCLUSIONS:**

Based on genetic evidence, smoking may be a risk factor for CTS. Further clinical trials are needed to confirm this causal relationship.

## INTRODUCTION

Carpal tunnel syndrome (CTS) is a nerve entrapment disorder primarily characterized by numbness, tingling, and weakness in the fingers due to compression of the median nerve within the carpal tunnel. Its prevalence in the general population is approximately 8.0%^1,2^. Potential pathophysiological mechanisms include elevated pressure within the carpal tunnel, ischemic changes in the median nerve, and compression from adjacent structures^3,4^. Risk factors for CTS include age, gender, obesity, manual labor, diabetes, and hypothyroidism^5-7^. Smoking is considered a contentious risk factor for CTS, with current debate surrounding its role^8,9^.

Smoking is a risk factor for multiple diseases, including respiratory diseases, cardiovascular diseases, liver and kidney dysfunction, and skeletal disorders^10-12^. The mechanisms through which smoking affects health are complex. For long-term smokers, whether active or passive, harmful substances may induce various phenotypic changes and functional impairments in macrophages, endothelial cells, and smooth muscle cells through various mechanisms^13^. This can promote the occurrence and progression of vascular diseases. Smoking is associated with reduced blood supply, oxidative stress, and decreased systemic inflammation, which may predispose peripheral nerves to compression neuropathies^14,15^. A birth cohort study involving 8703 individuals suggested an association between personal smoking history and CTS^16^. However, meta-analyses have not consistently supported a link between smoking and CTS, suggesting that observed associations may be due to confounding factors^9^.

Mendelian randomization (MR) is an effective method for inferring causal relationships between exposures and outcomes, based on Mendel’s laws of inheritance and instrumental variable estimation^17^. MR utilizes genetic variants as instrumental variables, allowing it to overcome confounding biases and be less susceptible to traditional study design limitations. Previous MR analyses have identified obesity and diabetes as risk factors for CTS^18^. However, there has been a lack of MR analysis investigating the relationship between smoking and CTS. The aim of this study is to explore whether smoking has an impact on CTS using the MR method.

## METHODS

### Study design and data sources

This study was a secondary analysis of publicly available GWAS data. The GWAS data for four smoking-related behaviors were used as exposures: smoking initiation, smoking status, lifetime smoking, and never smoking. Smoking initiation means regular smoking (current or former)^19^. The GWAS data for smoking status were sourced from a study involving 468170 individuals of European descent, published in 2018^20^. The GWAS of lifetime smoking comes from an article published in 2019, which combined smoking measurements with a simulated half-life constant to obtain a lifetime smoking index^21^. GWAS data for never smoking were obtained from the UK Biobank. The CTS data for the discovery set were extracted from meta-analyses conducted in 2021^22^. The CTS data for the validation set were sourced from the R10 version of the FinnGen database. The diagnostic code for CTS was ICD-9-3540 or ICD-10-G56.0. The discovery set is the sample dataset used for preliminary analysis and exploration. Its main purpose is to investigate whether smoking increases the risk of developing CTS. The validation set is used to verify whether the results from the discovery set are generalizable and robust. There was no significant sample overlap between GWAS for exposures and outcomes. Detailed information regarding the GWAS data is provided in Table 1.

**Table 1:** Basic information about the GWAS data used in this study

*GWAS*	*Sample size*	*Number of SNPs*	*Population*	*Year*
Smoking status	468170	11973425	European	2018
Never smoking	359706	13586591	European	2018
Smoking initiation	607291	11802365	European	2019
Lifetime smoking	502647	7693354	European	2019
CTS (Discovery set)	480201	24181062	European	2021
CTS (Validation set)	385304	21305860	European	2023

CTS: carpal tunnel syndrome. GWAS: genome-wide association study. SNP: single nucleotide polymorphism.

### Instrument variable selection

**Figure 1:**
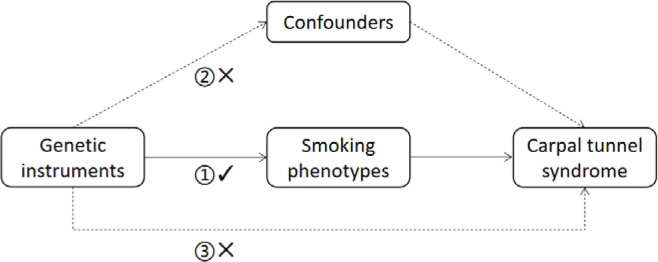
Schematic diagram of the three assumptions in Mendelian randomization analysis: 1) Association assumption: The instrumental variable is strongly associated with the exposure; 2) Independence assumption: The instrumental variable is independent of confounders; 3) Exclusion assumption: The instrumental variable affects the outcome only through the exposure

In accordance with the three major assumptions of MR analysis (Figure 1), eligible single nucleotide polymorphisms (SNPs) were selected based on the following criteria:

1. Genome-wide significance: SNPs associated with smoking traits should demonstrate genome-wide significance (p<5×10^-8^), referencing data from the European 1000 Genomes Project.
2. Physical distance and linkage disequilibrium: SNPs should have a physical distance greater than 10000 kb between them, and the LD threshold between genes should be less than an r^2^ value of 0.001.
3. Removal of palindromic alleles: Palindromic alleles were excluded.
4. PhenoScanner database: SNPs associated with potential confounding factors related to CTS were removed using the PhenoScanner database. We excluded SNPs associated with BMI (rs9835772, rs6265, etc.), type 2 diabetes (rs62107261), risk-taking behavior (rs326341, rs12244388, etc.), and trauma (rs3896224)^18,23^.
5. F-statistic threshold: SNPs with an F-statistic less than 10 were excluded. A low F-statistic suggests the presence of weak instrument bias, which may affect the results. The F-statistic for a single SNP is calculated^24,25^ as F=(β/SE)^2^.

### Statistical analysis

The main method used in this study is the inverse variance weighted (IVW) method with a fixed-effects model. This method does not consider the intercept term in the regression process, but instead uses the inverse of the result variance as weights for fitting^26^. Τhe beta values and 95% CI outputted by IVW are converted into OR, i.e. OR=e^β^. Additionally, MR-Egger regression and Weighted Median methods were utilized as supplementary analyses^27^. MR-Egger regression is based on the assumption of instrument strength independent of direct effect (InSIDE). Finally, a meta-analysis was conducted on the MR results (IVW) for both the discovery and validation sets. Depending on the presence of heterogeneity, either a random-effects model (in cases of heterogeneity) or a fixed-effects model (in the absence of heterogeneity) was applied.

### Sensitivity analysis

The MR-Presso method was employed to detect outliers. If outliers were identified, they were removed, and the analysis was repeated. Sensitivity analysis using the ‘leave-one-out’ method involved iteratively removing one SNP at a time to assess whether specific variants were driving the association between exposure and outcome variables. Furthermore, to ascertain the presence of horizontal pleiotropy in MR analysis, the MR-Egger intercept test was conducted. If the intercept term in the MR-Egger analysis yielded statistically significant results (p<0.05), it indicated significant horizontal pleiotropy. Finally, the Cochran’s Q statistic was used to test for heterogeneity. A statistically significant result in the Cochran’s Q statistic test (p<0.05) indicated heterogeneity in the analysis^28^. These tests and sensitivity analyses were conducted to ensure the robustness and validity of the MR results^29^.

**Figure 2:**
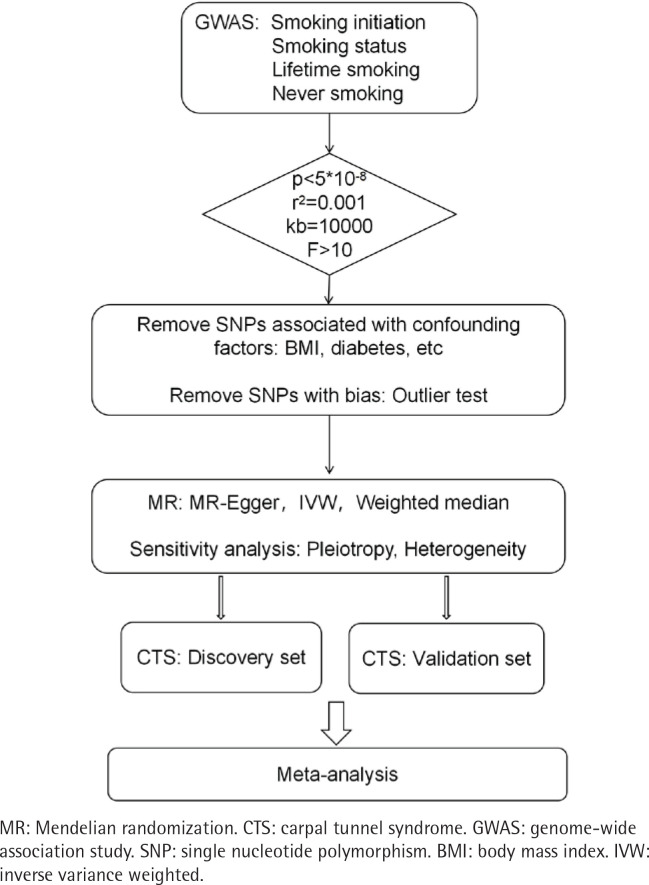
Flowchart of the impact of four smoking phenotypes on CTS through MR analysis

In this study, correlations with a p<0.05 were considered statistically significant. The analyses were conducted using R packages, including TwoSampleMR (0.5.8), *ieugwasr* (0.2.1), *metafor* (4.6.0) and MRPRESSO (1.0). The MR analysis diagram is shown in Figure 2.

We have adhered to the STROBE-MR guidelines and have included the STROBE-MR checklist (Supplementary file)^[Bibr CIT0030]30^.

## RESULTS

**Figure 3:**
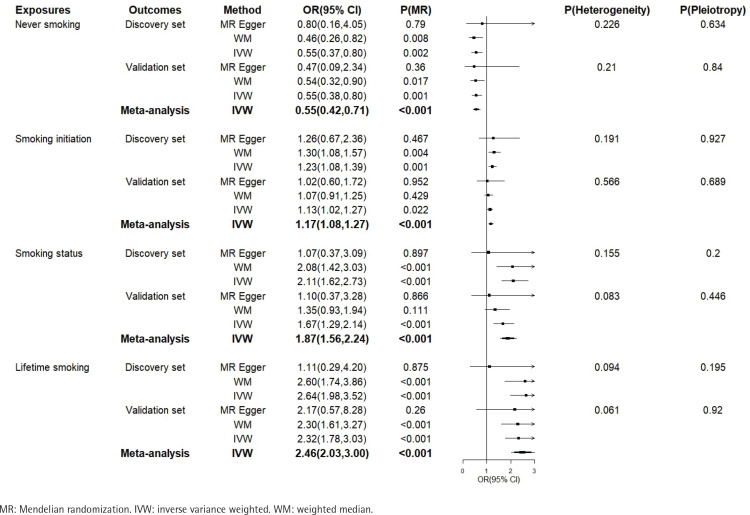
Forest plot of results of MR analysis on the impact of four smoking phenotypes on carpal tunnel syndrome

IVW results show that smoking initiation, smoking status, and lifetime smoking are risk factors for CTS in both the discovery and validation sets. The meta-analysis summary results are as follows: smoking initiation (OR=1.17; 95% CI: 1.08–1.27, p<0.001), smoking status (OR=1.87; 95% CI: 1.56–2.24, p<0.001), and lifetime smoking (OR=2.46; 95% CI: 2.03–3.00, p<0.001). MR-Egger and Weighted Median results also indicated a positive association between smoking and CTS (Figure 3).

Conversely, never smoking is a protective factor against CTS. The meta-analysis summary result for this is: OR=0.55; 95% CI: 0.42–0.71, p<0.001. MR-Egger and Weighted Median results also indicated a positive association between smoking and CTS (Figure 3). Since there was no heterogeneity in the above meta-analyses, a fixed-effects model was used. Sensitivity analysis indicated no significant heterogeneity or pleiotropy in our MR analysis. ‘Leaveone-out’ analysis demonstrated that individual SNPs did not substantially influence the results, suggesting the robustness of our MR analysis. The detailed information about the SNPs and the ‘leave-one-out’ method plot are provided in the Supplementary file.

## DISCUSSION

In this MR analysis, the four smoking phenotypes – smoking initiation, smoking status, lifetime smoking, and never smoking – serve as mutual validations, leading to the conclusion that smoking may be a risk factor for CTS.

Smoking is known to reduce blood circulation and induce nerve fibrosis^31,32^. Nicotine and carbon monoxide, two hazardous substances found in cigarette smoke, cause oxidative stress, endanger arterial walls, and accelerate the accumulation of fat plaques in blood vessels. Additionally, carbon monoxide in smoke decreases the blood’s oxygen-carrying capacity^15^. Smoking cessation enhances the function of high-density lipoprotein and cholesterol efflux, thereby reducing the risk of plaque formation^33^. Moreover, the mechanical damage caused by smoking posture and repetitive wrist flexion may induce fibrosis in the median nerve, impede its movement within the carpal tunnel, though further research is needed to confirm this mechanical effect.

Research on the association between smoking and CTS is limited and yields inconsistent results. A study investigating the impact of smoking on the outcomes of open carpal tunnel release surgery found that smokers had higher preoperative and postoperative QuickDASH scores, indicating the harmful effects of smoking on CTS^34^. However, a meta-analysis in 2022 incorporating 13 cross-sectional studies, 10 case-control studies, and 8 cohort studies, showed only one cross-sectional study demonstrating an association between smoking and CTS. The observed association in cross-sectional studies may be influenced by confounding factors^35^. This inconsistency with our study’s results could be attributed to the lower quality of literature included in the meta-analysis.

### Strengths and limitations

Compared to observational studies, our study has significant advantages. By utilizing MR, we employed genetic information as instrumental variables for causal inference, providing statistically convincing results. Additionally, the large sample size of our study surpasses previous research capabilities. However, limitations exist due to constraints of the GWAS data; we could not consider gender factors. The GWAS used in this study are based on European samples, so the MR results may not be applicable to other ethnic groups. Furthermore, our study cannot address unobserved pleiotropy, thus results may be subject to some degree of bias. It should be noted that the causal relationship identified in this study is based on genetic evidence, and further clinical trials are needed to validate this relationship. Additionally, randomized controlled trials are needed to explore whether individuals with smoking-related SNP characteristics but who never smoke still have a risk of developing CTS. Therefore, the conclusions of this MR study should be interpreted with caution.

## CONCLUSIONS

This MR study provides genetic evidence supporting smoking as a potential risk factor for CTS, with smoking initiation, smoking status, and lifetime smoking all showing significant associations with CTS. Conversely, never smoking appears to be a protective factor against CTS. These findings suggest that smoking cessation could be an effective preventive measure for CTS.

## Supplementary Material



## Data Availability

The data supporting this research are available from the following sources: The GWAS data for Smoking initiation, Smoking status, Never smoked, and CTS (discovery set) can be downloaded from the IEU database (https://gwas.mrcieu.ac.uk/datasets/) with the registration numbers ieu-b-4877, ebi-a-GCST90029014, ukb-d-20116_0, ebi-a-GCST90018813. Lifetime smoking GWAS summary data are available for download at https://doi.org/10.5523/bris.10i96zb8gm0j81yz0q6ztei23d. CTS (validation) data in the FinnGen database download: https://storage.googleapis.com/finngen-public-data-r10/summary_stats/finngen_R10_G6_CARPTU.gz
