# Supplementary material for: Smoking may be a risk factor for carpal tunnel syndrome: Insights from Mendelian randomization analysis
Source: Tob Induc Dis. 2025 Jan 30;23:10.18332/tid/199930. doi: 10.18332/tid/199930 (PMC11780312; doi:10.18332/tid/199930)
Supplement: Supplementary file 1 [file TID-23-09-s1.pdf]

Instrument Variables for MR analysis of smoking initiation and carpal tunnel syndrome

SNP	beta.exposure	se.exposure	p.exposure	beta.outcome	se.outcome	p.outcome
rs12025237	-0.033	0.005339	6.52E-10	-0.011	0.0152	0.4704
rs2046850	-0.02481	0.004478	3.03E-08	0.0169	0.0132	0.199
rs3001723	0.033512	0.003898	8.12E-18	-0.0031	0.012	0.7993
rs6669839	0.026004	0.004396	3.36E-09	0.0111	0.0135	0.411
rs301805	0.021468	0.003613	2.80E-09	-0.0167	0.0112	0.137
rs12042107	-0.02228	0.003568	4.22E-10	-0.0056	0.011	0.6118
rs10905461	-0.02396	0.004145	7.35E-09	0.0035	0.0132	0.7923
rs7938812	0.043791	0.003637	2.71E-33	0.0141	0.0113	0.2096
rs6265	-0.03179	0.004578	3.77E-12	0.0097	0.0142	0.4953
rs4523689	-0.02061	0.003643	1.55E-08	0.0074	0.0111	0.5008
rs7929518	0.024238	0.004285	1.56E-08	-0.0137	0.0128	0.2865
rs1971318	0.028507	0.004925	7.06E-09	0.0055	0.0156	0.7238
rs11057005	-0.02093	0.003579	4.85E-09	-0.0055	0.011	0.6187
rs7322872	-0.02557	0.004335	3.58E-09	0.0016	0.0163	0.9238
rs3904512	-0.02116	0.003577	3.23E-09	0	0.011	0.9981
rs76214862	-0.02499	0.004547	3.99E-08	-0.004	0.0135	0.7658
rs1435741	0.029415	0.003591	2.64E-16	0.0024	0.0111	0.8315
rs12441907	-0.02921	0.004523	1.06E-10	0.0093	0.014	0.5043
rs4781977	-0.02387	0.004365	4.54E-08	-0.0079	0.0145	0.5853
rs4785836	-0.02047	0.003659	2.26E-08	-0.0037	0.0112	0.7407
rs1050847	-0.02162	0.003589	1.67E-09	-0.018	0.0111	0.1031
rs11658881	0.020136	0.003611	2.43E-08	-4.00E-04	0.0111	0.972
rs7224742	-0.02071	0.003655	1.43E-08	-0.0022	0.0113	0.8488
rs11872397	-0.02477	0.004095	1.43E-09	0.0013	0.0128	0.9205
rs76608582	-0.04956	0.00826	1.94E-09	0.0088	0.0256	0.7322
rs266047	-0.03051	0.003739	3.36E-16	-0.0115	0.0109	0.2917
rs13030994	0.036093	0.003556	3.56E-24	0.0096	0.0109	0.3789
rs1445649	0.023993	0.003565	1.68E-11	0.0161	0.0109	0.1395
rs6433897	0.022448	0.004058	3.16E-08	-7.00E-04	0.0124	0.958
rs4674993	-0.02521	0.004436	1.32E-08	0.0051	0.0138	0.7147
rs134529	-0.01998	0.003661	4.85E-08	-0.01	0.0113	0.3747
rs1154693	0.032622	0.004912	3.12E-11	0.0118	0.0146	0.4177
rs12632110	-0.02338	0.003753	4.78E-10	-0.0238	0.0118	0.04316
rs1869243	0.019741	0.003563	2.97E-08	0.0142	0.011	0.196
rs66680800	-0.02027	0.003653	2.83E-08	0.0138	0.0111	0.2143
rs10001365	-0.02499	0.003642	6.65E-12	-9.00E-04	0.0111	0.9319
rs962625	0.023718	0.004038	4.37E-09	0.0165	0.0128	0.1973
rs993700	-0.02593	0.004292	1.53E-09	-0.015	0.013	0.2509
rs12186738	-0.03326	0.005021	3.42E-11	-0.0031	0.0156	0.8415
rs72789632	-0.03289	0.005286	5.02E-10	0.0305	0.0173	0.07825
rs1385108	0.024662	0.004157	3.00E-09	0.0082	0.0125	0.5098
rs4044321	-0.02784	0.003711	6.08E-14	-0.0223	0.0114	0.05023

rs6893752	-0.0241	0.004074	3.25E-09	0.0149	0.0126	0.2346
rs4352629	-0.02753	0.003569	1.22E-14	-0.011	0.0109	0.3144
rs3800227	0.022812	0.004058	1.93E-08	-0.0167	0.0122	0.1717
rs9401770	0.027731	0.003986	3.47E-12	0.0162	0.012	0.1775
rs10233018	0.027069	0.003557	2.75E-14	-0.0184	0.011	0.09271
rs10279261	-0.02142	0.003663	5.00E-09	-0.0225	0.0113	0.04548
rs4236259	-0.02477	0.003557	3.35E-12	-0.0219	0.011	0.04649
rs10260968	-0.02032	0.003609	1.75E-08	-0.0051	0.011	0.6456
rs12112638	-0.02453	0.004043	1.34E-09	-0.0089	0.0123	0.468
rs3801289	-0.02206	0.00374	3.74E-09	-0.0289	0.0114	0.01084
rs12333760	-0.02905	0.004801	1.44E-09	0.0138	0.0141	0.327
rs1565735	-0.03762	0.004461	3.42E-17	-0.01	0.0141	0.4791
rs13261666	-0.02689	0.003556	3.90E-14	-0.0071	0.0109	0.5165
rs12545053	0.020281	0.003637	2.43E-08	0.0084	0.0111	0.4469
rs2631024	-0.02296	0.004028	1.18E-08	-0.0133	0.0122	0.2744
rs1899896	0.026448	0.003887	1.04E-11	0.0179	0.0121	0.1392
rs10114490	-0.02551	0.004532	1.81E-08	0.0089	0.0172	0.6032
rs4543592	0.021931	0.003562	7.46E-10	0.0214	0.011	0.05191
rs1004787	0.029923	0.003571	5.26987E-17	0.0317	0.0111	0.004286
rs2678897	0.020087	0.003643	3.51002E-08	-0.0162	0.0112	0.1484

Instrument Variables for MR analysis of smoking status and carpal tunnel syndrome

SNP	beta.exposure	se.exposure	p.exposure	beta.outcome	se.outcome	p.outcome
rs11802893	-0.01431	0.002108	1.20E-11	-0.0047	0.0157	0.7636
rs10863810	-0.01008	0.001666	1.10E-09	0.009	0.0127	0.4791
rs34000440	-0.00947	0.001671	2.10E-08	-0.0074	0.0135	0.5817
rs4949465	0.011307	0.002039	1.80E-08	0.0321	0.0156	0.03938
rs653953	-0.011	0.001463	3.10E-14	0.0023	0.0116	0.8456
rs10458563	0.011207	0.00169	2.10E-11	0.0108	0.0135	0.422
rs12042444	-0.0096	0.001421	1.30E-11	0.0312	0.0113	0.005777
rs301805	0.008032	0.001392	1.40E-08	-0.0167	0.0112	0.137
rs2391706	-0.00779	0.001389	2.30E-08	-0.0089	0.011	0.4191
rs1926030	0.013745	0.001408	1.20E-22	0.0038	0.0112	0.7328
rs12770682	0.010167	0.001826	2.60E-08	-0.0088	0.0148	0.5553
rs9423279	-0.0099	0.001474	2.00E-11	-0.0051	0.0115	0.6609
rs2588978	-0.01063	0.001373	7.50E-15	-0.0218	0.0109	0.04534
rs2175207	0.011637	0.001875	4.10E-10	0.0184	0.0143	0.1979
rs11255887	0.010037	0.001592	3.10E-10	-0.006	0.0132	0.6466
rs2155646	0.019494	0.00141	2.80E-43	0.0139	0.0113	0.2179
rs2010921	0.009554	0.001484	9.80E-11	0.0122	0.0117	0.297
rs56213534	-0.00942	0.001496	2.40E-10	-0.0335	0.0118	0.004652

rs35891966	-0.01505	0.002666	1.40E-08	0.0189	0.0208	0.3636
rs11066972	-0.01144	0.001982	9.70E-09	-5.00E-04	0.0157	0.9742
rs1109480	-0.00885	0.00142	4.50E-10	0.0079	0.0113	0.4865
rs7977812	0.008841	0.0016	3.30E-08	0.0171	0.0125	0.1712
rs7333559	-0.00991	0.001694	3.60E-09	-0.0103	0.0138	0.456
rs9576071	-0.00749	0.001375	4.70E-08	-0.0077	0.0109	0.4784
rs12433109	0.008523	0.001409	2.50E-09	-0.0105	0.0111	0.3458
rs7155595	0.00811	0.001472	3.90E-08	0.0137	0.0118	0.2443
rs4899753	0.013409	0.00242	4.00E-08	0.0272	0.0196	0.1649
rs1381274	0.008068	0.001376	4.90E-09	-0.0016	0.0109	0.8828
rs12910916	0.014039	0.001683	3.00E-17	0.0262	0.0133	0.04864
rs2017500	0.008973	0.001382	1.00E-10	0.0093	0.0109	0.3945
rs9646259	0.00855	0.001512	1.80E-08	0.0084	0.0121	0.4885
rs752894	0.008202	0.001471	2.50E-08	0.0218	0.0114	0.05628
rs3748387	0.00952	0.001438	4.80E-11	-0.0087	0.0115	0.4518
rs1050847	-0.00755	0.001396	4.80E-08	-0.018	0.0111	0.1031
rs4790874	0.008631	0.001383	4.70E-10	0.0292	0.0109	0.007546
rs1825733	-0.01137	0.001916	4.40E-09	-0.0036	0.0154	0.817
rs12970816	0.008664	0.001409	9.10E-10	0.0206	0.0112	0.06538
rs17733784	-0.00887	0.001425	6.50E-10	-0.0042	0.0112	0.7055
rs71367545	0.010511	0.001691	4.10E-10	0.0232	0.0133	0.08161
rs76608582	-0.02397	0.003409	1.20E-12	0.0088	0.0256	0.7322
rs1427506	-0.01195	0.00201	3.50E-09	0.0055	0.0153	0.7195
rs6433901	0.009353	0.001584	3.50E-09	0.0026	0.0127	0.8397
rs10210512	0.007527	0.00139	4.70E-08	0.009	0.011	0.4141
rs56059523	0.012049	0.002101	6.20E-09	0.0198	0.0162	0.2222
rs2339515	0.008728	0.001414	6.10E-10	0.0059	0.0111	0.5946
rs7572027	0.010922	0.001758	9.70E-10	-0.0085	0.0142	0.5488
rs11692742	0.011653	0.001543	2.20E-14	0.0305	0.012	0.01132
rs512942	0.010531	0.001404	5.60E-14	0.0065	0.0113	0.5677
rs542883	0.011275	0.001384	3.50E-16	0.0231	0.0112	0.03849
rs12465974	-0.00864	0.001431	7.90E-10	-0.0047	0.0113	0.6739
rs72804548	0.015534	0.002589	3.10E-09	-0.0122	0.0179	0.4951
rs3790283	-0.00817	0.001456	1.60E-08	-0.0293	0.0114	0.01027
rs77217252	0.011709	0.002067	1.60E-08	0.0359	0.0165	0.02977
rs762995	-0.00861	0.001378	4.00E-10	-0.0156	0.0109	0.1534
rs705219	0.016356	0.002174	4.30E-14	0.0153	0.0157	0.3312
rs9842947	0.008854	0.001467	2.20E-09	-0.0065	0.0117	0.5808
rs12487411	-0.00815	0.001376	3.60E-09	0.0029	0.0109	0.7928
rs4479577	0.00796	0.00138	1.20E-08	0.0138	0.011	0.2085
rs4676964	0.008445	0.001384	7.40E-10	0.0036	0.011	0.7471
rs34495106	-0.01272	0.001433	1.30E-18	-0.0297	0.0117	0.01102
rs12485709	-0.00987	0.00141	2.70E-12	0.014	0.0112	0.2131
rs72678859	-0.01241	0.001828	1.20E-11	-0.02	0.0149	0.1811

rs72712556	-0.00898	0.00147	2.30E-09	-0.0113	0.0116	0.329
rs7668995	-0.01207	0.001512	7.50E-16	0.0109	0.012	0.362
rs10461104	0.007848	0.001431	3.10E-08	0.001	0.0113	0.9277
rs17003752	-0.0112	0.002003	1.70E-08	0.0203	0.0165	0.2178
rs72886316	0.012216	0.002001	9.80E-10	-0.0117	0.0173	0.4996
rs4957528	0.009908	0.001708	2.90E-09	-0.0135	0.0142	0.3399
rs986391	-0.01232	0.001425	5.00E-18	-0.0205	0.0113	0.0706
rs12517438	0.007473	0.001381	4.90E-08	0.0165	0.011	0.1337
rs1559278	-0.0082	0.001432	1.20E-08	-0.0171	0.0116	0.1398
rs6893752	-0.00954	0.00157	9.30E-10	0.0149	0.0126	0.2346
rs73123076	0.008699	0.001429	2.20E-09	-0.0161	0.0138	0.2423
rs27003	0.009107	0.001497	8.50E-10	0.0051	0.0115	0.6573
rs9487626	-0.01654	0.001776	1.40E-20	-0.0223	0.0132	0.09256
rs3818987	-0.00858	0.001386	5.90E-10	-0.0105	0.0111	0.3459
rs12213996	0.007853	0.001411	4.40E-08	0.0178	0.0113	0.1138
rs4839955	-0.00818	0.001375	5.00E-09	-0.0103	0.011	0.3494
rs4730682	-0.00978	0.00138	1.10E-12	0.0107	0.0109	0.3303
rs6951574	0.009075	0.001389	7.70E-11	-0.0061	0.0135	0.6525
rs896777	0.007663	0.001395	3.30E-08	0.0062	0.011	0.5742
rs6963853	0.008754	0.001392	3.80E-10	0.005	0.011	0.6494
rs2705608	-0.00966	0.001433	1.60E-11	-0.0195	0.0115	0.08911
rs12333760	-0.011	0.001854	2.70E-09	0.0138	0.0141	0.327
rs1565735	-0.01881	0.001721	5.00E-28	-0.01	0.0141	0.4791
rs13263909	-0.00807	0.001475	4.30E-08	0.0061	0.0115	0.5951
rs12545435	-0.00827	0.001396	3.70E-09	-0.0022	0.011	0.8406
rs1899896	0.008939	0.001509	1.80E-09	0.0179	0.0121	0.1392
rs4837631	-0.00821	0.001379	1.80E-09	0.0092	0.0109	0.398
rs13301073	0.009935	0.001424	1.80E-12	0.0095	0.0113	0.3987
rs3025316	0.016286	0.002161	4.50E-14	-0.009	0.0196	0.6449
rs11103667	0.010263	0.001757	5.70E-09	0.024	0.0142	0.09147
rs1490320	-0.01295	0.002239	6.40E-09	0.0212	0.0183	0.247
rs1246292	0.009025	0.001476	1.50E-09	0.0114	0.0118	0.3331

Instrument Variables for MR analysis of lifetime smoking and carpal tunnel syndrome

SNP	beta.exposure	se.exposure	p.exposure	beta.outcome	se.outcome	p.outcome
rs10918701	0.008024	0.001432	2.10E-08	0.014	0.0113	0.2119
rs4949465	-0.01161	0.002058	1.70E-08	0.0321	0.0156	0.03938
rs549845	0.011261	0.001509	8.30E-14	0.0081	0.012	0.4971
rs1931263	-0.00761	0.001386	4.00E-08	0.012	0.011	0.2728
rs7519626	0.00842	0.001479	1.20E-08	0.006	0.0114	0.6012
rs7077678	0.00855	0.001436	2.60E-09	0.0025	0.0112	0.8237

rs2675638	0.008499	0.0014	1.30E-09	-0.0172	0.0111	0.1204
rs11255908	-0.01007	0.001588	2.30E-10	-0.004	0.0132	0.7591
rs17553262	-0.01273	0.002181	5.30E-09	0.0072	0.0182	0.6902
rs9919670	-0.01524	0.001421	7.60E-27	0.0137	0.0112	0.2233
rs34866095	-0.00857	0.001506	1.20E-08	0.0027	0.0122	0.8215
rs75742406	0.009619	0.001585	1.30E-09	-0.0236	0.0123	0.05478
rs17309874	-0.01129	0.001582	9.70E-13	0.0115	0.0124	0.3546
rs4391802	0.010319	0.001528	1.40E-11	-0.0197	0.0122	0.107
rs7297175	-0.00812	0.001399	6.60E-09	0.0188	0.011	0.08694
rs10879871	-0.00958	0.001458	5.00E-11	0.0016	0.0114	0.8882
rs12831617	-0.00918	0.001633	1.90E-08	0.0196	0.0134	0.1446
rs7333559	0.01074	0.001707	3.20E-10	-0.0103	0.0138	0.456
rs860326	0.008338	0.001402	2.70E-09	-0.0157	0.011	0.1524
rs7155595	-0.00885	0.001485	2.50E-09	0.0137	0.0118	0.2443
rs35175834	-0.0164	0.001698	4.60E-22	0.0269	0.0133	0.0429
rs28485305	0.008007	0.001439	2.60E-08	-0.014	0.0112	0.2144
rs8042134	-0.00994	0.001401	1.30E-12	0.0147	0.011	0.1794
rs6598539	-0.00815	0.001389	4.50E-09	0.0131	0.0109	0.2284
rs12708665	-0.00909	0.001539	3.50E-09	-0.0204	0.0121	0.09259
rs57611503	0.007743	0.00141	4.00E-08	-0.0191	0.0112	0.08778
rs60952428	0.013411	0.002419	3.00E-08	0.0191	0.0188	0.3106
rs1050847	0.00797	0.001405	1.40E-08	-0.018	0.0111	0.1031
rs369230	-0.00909	0.001511	1.80E-09	0.008	0.0117	0.4941
rs8614	-0.01146	0.001797	1.80E-10	0.01	0.0144	0.4876
rs732083	0.008348	0.001473	1.50E-08	-0.0056	0.0116	0.6298
rs9904288	0.008428	0.001523	3.10E-08	-0.0184	0.0121	0.1278
rs67596067	-0.00888	0.001458	1.20E-09	0.0059	0.0116	0.6143
rs12967855	0.008189	0.001479	3.10E-08	0.0023	0.0118	0.8468
rs62098013	-0.00857	0.001457	4.10E-09	0.0343	0.0115	0.002798
rs71367545	-0.01032	0.001704	1.40E-09	0.0232	0.0133	0.08161
rs35343344	0.009181	0.001596	8.80E-09	-0.0047	0.0127	0.7114
rs76608582	0.021632	0.00344	3.20E-10	0.0088	0.0256	0.7322
rs62155874	-0.01691	0.002085	5.20E-16	0.0126	0.0168	0.456
rs3811038	-0.00954	0.001557	8.90E-10	0.0127	0.0125	0.3092
rs62175972	0.021746	0.003857	1.70E-08	0.0063	0.0334	0.8502
rs13009008	0.008633	0.001473	4.60E-09	-0.0139	0.0115	0.2246
rs12623702	-0.00977	0.001428	7.70E-12	0.0182	0.0111	0.1015
rs13016665	-0.00849	0.001412	1.80E-09	-0.0114	0.011	0.2997
rs4814873	0.009712	0.001636	2.90E-09	-0.031	0.0128	0.01552
rs6119897	-0.0128	0.001627	3.60E-15	-0.0043	0.0124	0.7271
rs348809	-0.00828	0.001456	1.30E-08	0.0155	0.0115	0.1791
rs6011779	0.019115	0.001764	2.30E-27	-0.0111	0.0135	0.4106
rs147412694	-0.01157	0.001949	2.90E-09	0.0386	0.0156	0.01362
rs2838834	-0.00936	0.001515	6.30E-10	0.0018	0.0121	0.8818

rs136233	-0.00996	0.001769	1.80E-08	0.0165	0.014	0.239
rs326341	0.009435	0.001392	1.20E-11	0.0042	0.0109	0.7039
rs73220544	-0.01082	0.001913	1.50E-08	0.028	0.0141	0.04728
rs9842947	-0.00877	0.001481	3.10E-09	-0.0065	0.0117	0.5808
rs6779302	-0.00875	0.001439	1.20E-09	0.0157	0.0113	0.1658
rs6778080	0.011114	0.001566	1.30E-12	-0.0215	0.0132	0.1025
rs72678864	0.012383	0.001839	1.60E-11	-0.0204	0.0149	0.1699
rs17576594	0.01095	0.001552	1.70E-12	0.0037	0.0121	0.7616
rs624833	0.009286	0.001504	6.60E-10	-0.0209	0.0117	0.07517
rs4957528	-0.01012	0.001722	4.20E-09	-0.0135	0.0142	0.3399
rs11948770	-0.01024	0.001645	4.90E-10	-0.0178	0.0127	0.1606
rs986391	0.011139	0.001438	9.40E-15	-0.0205	0.0113	0.0706
rs71627581	0.013257	0.002199	1.60E-09	-0.0084	0.0162	0.6048
rs10052591	0.0084	0.001402	2.10E-09	-0.02	0.0112	0.0731
rs4571506	0.007877	0.001392	1.50E-08	-0.0106	0.0109	0.3306
rs7766610	0.012584	0.001793	2.20E-12	-0.0227	0.0132	0.08699
rs6935954	0.009582	0.001402	8.20E-12	0.0073	0.0112	0.5182
rs2254710	0.008996	0.001631	3.50E-08	-0.005	0.0123	0.6875
rs12202536	-0.00823	0.001386	2.80E-09	0.0229	0.0109	0.0357
rs10282292	0.008962	0.001447	5.90E-10	-0.0339	0.0113	0.002714
rs7807019	-0.01042	0.001391	6.70E-14	-0.0168	0.011	0.1263
rs6957896	-0.00758	0.001387	4.50E-08	-0.0082	0.011	0.4574
rs4731925	-0.00829	0.00149	2.60E-08	0.0039	0.0118	0.7392
rs10226228	-0.01141	0.001437	2.00E-15	0.0111	0.0115	0.3374
rs1922018	0.010033	0.001438	3.00E-12	-0.0213	0.0114	0.06063
rs11768481	0.009012	0.001475	9.90E-10	-0.0283	0.0142	0.0458
rs6962772	0.011064	0.001916	7.80E-09	0.0196	0.0142	0.1684
rs11783093	0.01571	0.001897	1.20E-16	-0.0131	0.0154	0.3955
rs2062882	-0.00811	0.00142	1.10E-08	0.0118	0.0111	0.2879
rs13296519	-0.0097	0.001419	8.10E-12	0.019	0.0112	0.08902
rs4543592	-0.00866	0.001389	4.50E-10	0.0214	0.011	0.05191
rs7039819	0.008734	0.001405	5.10E-10	-0.0185	0.011	0.09164
rs1246265	-0.00887	0.001509	4.20E-09	0.0062	0.0119	0.6004

Instrument Variables for MR analysis of never smoking and carpal tunnel syndrome

SNP	beta.exposure	se.exposure	p.exposure	beta.outcome	se.outcome	p.outcome
rs12745098	0.011481	0.001779	1.09E-10	-0.0025	0.0205	0.9039
rs6676022	0.011593	0.001779	7.19E-11	-0.0045	0.0157	0.773
rs55921136	0.008595	0.001448	2.93E-09	0.0136	0.0131	0.2973
rs12725407	0.008139	0.001479	3.72E-08	-0.0101	0.0142	0.4747
rs1143702	0.008118	0.001239	5.62E-11	0.0022	0.0116	0.8496
rs6690680	0.008841	0.001597	3.08E-08	-0.0032	0.0146	0.8249
rs299688	-0.00727	0.001312	2.95E-08	0.0053	0.0125	0.67

rs9423279	0.00767	0.001246	7.58E-10	-0.0051	0.0115	0.6609
rs2675628	0.007109	0.00116	8.82E-10	-0.0216	0.0109	0.04735
rs1899888	0.007296	0.001163	3.54E-10	-0.0164	0.0109	0.1339
rs10905461	0.007273	0.001332	4.76E-08	0.0035	0.0132	0.7923
rs7948789	-0.01617	0.001197	1.33E-41	0.0127	0.0113	0.2601
rs523528	0.008071	0.001192	1.30E-11	-0.0022	0.0111	0.845
rs35891966	0.014775	0.002244	4.56E-11	0.0189	0.0208	0.3636
rs4910656	0.006844	0.001228	2.47E-08	-0.0165	0.0116	0.1542
rs2867748	0.006942	0.001247	2.59E-08	-0.0113	0.0117	0.3351
rs10897561	-0.00669	0.001223	4.50E-08	-0.005	0.0113	0.6605
rs379525	-0.00649	0.00119	4.96E-08	0.0048	0.0111	0.6667
rs772921	0.007273	0.001223	2.74E-09	-0.0151	0.0116	0.192
rs7333559	0.008052	0.001436	2.05E-08	-0.0103	0.0138	0.456
rs1027460	0.007082	0.001186	2.38E-09	-0.0018	0.0112	0.8746
rs12910916	-0.00901	0.00142	2.17E-10	0.0262	0.0133	0.04864
rs11631530	-0.00999	0.001798	2.77E-08	-0.017	0.0165	0.3022
rs16951001	-0.0066	0.001179	2.16E-08	0.0102	0.0111	0.3611
rs11646575	-0.00824	0.001172	2.00E-12	0.0091	0.011	0.4076
rs12450028	-0.00706	0.001221	7.32E-09	-0.0091	0.0115	0.4306
rs4566215	0.006622	0.001172	1.62E-08	-0.0097	0.0111	0.3826
rs12608052	0.006754	0.001164	6.48E-09	-0.0044	0.0109	0.6885
rs76608582	0.018289	0.00286	1.61E-10	0.0088	0.0256	0.7322
rs12479064	-0.00804	0.001468	4.36E-08	0.0046	0.014	0.7446
rs10193706	-0.01177	0.00117	8.25E-24	-0.0115	0.0112	0.303
rs6433897	-0.00724	0.00132	4.25E-08	-7.00E-04	0.0124	0.958
rs528301	-0.0086	0.001168	1.77E-13	0.0279	0.0111	0.01203
rs1029986	-0.00702	0.001195	4.27E-09	0.0237	0.0113	0.03614
rs6141314	-0.00806	0.001364	3.40E-09	-0.0037	0.0123	0.7662
rs1499970	-0.01093	0.001736	3.04E-10	0.0127	0.0152	0.4038
rs12487411	0.007505	0.001165	1.19E-10	0.0029	0.0109	0.7928
rs11917003	0.008967	0.001419	2.65E-10	0.0034	0.0134	0.8009
rs11127903	-0.00807	0.001355	2.59E-09	0.039	0.0129	0.002414
rs72678859	0.009708	0.001537	2.71E-10	-0.02	0.0149	0.1811
rs748828	0.008621	0.001289	2.26E-11	0.012	0.012	0.3208
rs6828849	0.006712	0.00118	1.28E-08	-0.0238	0.0112	0.03352
rs17003752	0.009861	0.0017	6.58E-09	0.0203	0.0165	0.2178
rs4957528	-0.00847	0.001446	4.58E-09	-0.0135	0.0142	0.3399
rs1549213	0.008527	0.001212	1.95E-12	-0.021	0.0114	0.06528
rs42417	-0.00703	0.001263	2.54E-08	0.0048	0.0115	0.6768
rs9487626	0.013103	0.001504	3.07E-18	-0.0223	0.0132	0.09256
rs9381910	0.011091	0.001918	7.34E-09	-0.0285	0.0218	0.19
rs7451586	-0.00667	0.001195	2.35E-08	0.02	0.0112	0.07472
rs9375371	-0.0074	0.001312	1.74E-08	0.0161	0.012	0.1811
rs10233018	-0.00766	0.001163	4.59E-11	-0.0184	0.011	0.09271

rs6466631	-0.00687	0.001163	3.52E-09	-0.0189	0.0109	0.08499
rs72505558	0.006744	0.001188	1.39E-08	-0.012	0.0137	0.3819
rs883403	0.009424	0.001607	4.55E-09	0.0208	0.0142	0.1439
rs17151637	0.007511	0.001292	6.12E-09	0.0061	0.0122	0.6167
rs1565735	0.01159	0.001458	1.87E-15	-0.01	0.0141	0.4791
rs10956804	0.006608	0.001171	1.69E-08	-0.0058	0.0109	0.5987
rs2416770	-0.00649	0.001167	2.71E-08	-0.0144	0.0134	0.2802
rs7870475	-0.00719	0.001163	6.33E-10	0.0011	0.0109	0.9187
rs11103667	-0.0086	0.001486	7.03E-09	0.024	0.0142	0.09147
rs117435980	-0.0092	0.001574	5.02E-09	-0.0204	0.0142	0.1503
rs1561195	-0.00789	0.001188	3.01E-11	0.0156	0.0137	0.2539

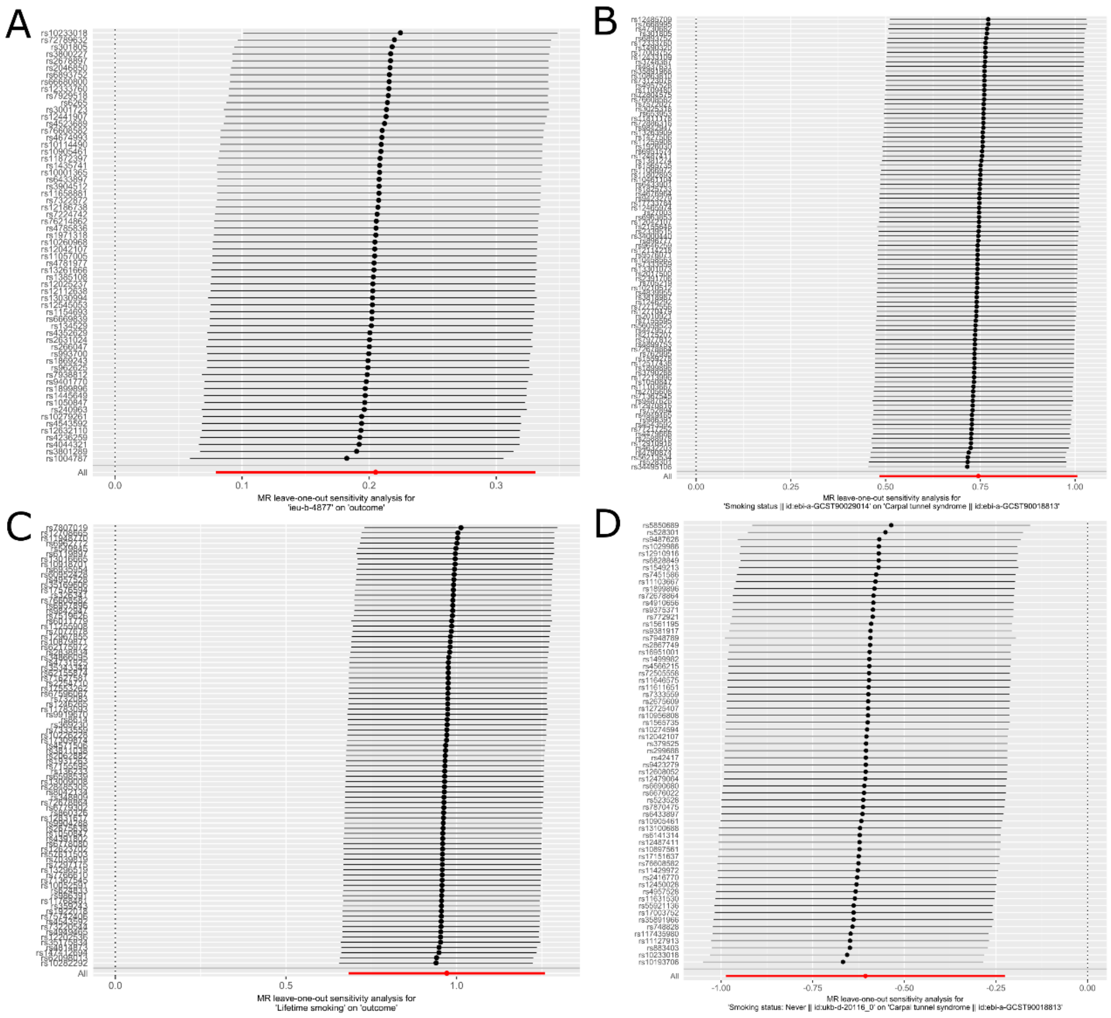

Leave-one-out. Sensitivity analysis of Mendelian randomization analysis on the relationship between four smoking phenotypes and CTS. A. Smoking initiation for CTS. B. Smoking status for CTS. C. Lifetime smoking for CTS. D. Never smoking for CTS. CTS: carpal tunnel syndrome.

STROBE-MR checklist of recommended items to address in reports of Mendelian randomization studies[1 2]

Item No.	Section		Checklist item	Page No.	Relevant text from manuscript
1	**TITLE and ABSTRACT**		Indicate Mendelian randomization (MR) as the study's design in the title and/or the abstract if that is a main purpose of the study	1	Smoking may be a Risk Factor for Carpal Tunnel Syndrome: Insights from Mendelian Randomization Analysis
	**INTRODUCTION**				
2	**Background**		Explain the scientific background and rationale for the reported study. What is the exposure? Is a potential causal relationship between exposure and outcome plausible? Justify why MR is a helpful method to address the study question	4,5	This can promote the occurrence and progression of vascular diseases. Smoking is associated with reduced blood supply, oxidative stress, and decreased systemic inflammation, which may predispose peripheral nerves to compression neuropathies<br>Mendelian randomization (MR) is an effective method for inferring causal relationships between exposures and outcomes, based on Mendel's laws of inheritance and instrumental variable estimation
3	**Objectives**		State specific objectives clearly, including pre-specified causal hypotheses (if any). State that MR is a method that, under specific assumptions, intends to estimate causal effects	5	However, there has been a lack of MR analysis investigating the relationship between smoking and CTS, which is the focus of the current study.
	**METHODS**				
4	**Study design and data sources**		Present key elements of the study design early in the article. Consider including a table listing sources of data for all phases of the study. For each data source contributing to the analysis, describe the following:		
		a)	Setting: Describe the study design and the underlying population, if possible. Describe the setting, locations, and relevant dates, including periods of recruitment, exposure, follow-up, and data collection, when available.	6	Detailed information regarding the GWAS data is provided in Table 1.
		b)	Participants: Give the eligibility criteria, and the sources and methods of selection of participants. Report the sample size, and whether any power or sample size calculations were carried out prior to the main analysis	6	Detailed information regarding the GWAS data is provided in Table 1.
		c)	Describe measurement, quality control and selection of genetic variants	6	In accordance with the three major assumptions of MR analysis, eligible single nucleotide polymorphisms (SNPs) were selected based on the following criteria: 1. Genome-Wide Significance: SNPs associated with smoking traits should demonstrate genome-wide significance (P < 5×10^-8), referencing data from the European 1000 Genomes Project. 2. Physical Distance and Linkage Disequilibrium: SNPs should have a physical distance

					greater than 10,000 kb between them, and the LD threshold between genes should be less than an r^2 value of 0.001. 3. Removal of Palindromic Alleles: Palindromic alleles were excluded. 4. PhenoScanner Database: SNPs associated with potential confounding factors related to CTS were removed using the PhenoScanner database. 5. F-Statistic Threshold: SNPs with an F-statistic less than 10 were excluded. A low F-statistic suggests the presence of weak instrument bias, which may affect the results. The F-statistic for a single SNP is calculated as F = (beta/se)^2
		d)	For each exposure, outcome, and other relevant variables, describe methods of assessment and diagnostic criteria for diseases	5,6	Smoking initiation means regular smoking (current or former). The GWAS for lifetime smoking comes from a MR analysis, which combined smoking measurements with a simulated half-life constant to obtain a lifetime smoking index. The ICD10 code for CTS is G56.0.
		e)	Provide details of ethics committee approval and participant informed consent, if relevant		NA
5	**Assumptions**		Explicitly state the three core IV assumptions for the main analysis (relevance, independence and exclusion restriction) as well assumptions for any additional or sensitivity analysis	6,7,8	In accordance with the three major assumptions (relevance, independence and exclusion restriction) of MR analysis, eligible single nucleotide polymorphisms (SNPs) were selected based on the following criteria<br><br>The MR-Presso method was employed to detect outliers. If outliers were identified, they were removed, and the analysis was repeated. Sensitivity analysis using the "leave-one-out" method involved iteratively removing one SNP at a time to assess whether specific variants were driving the association between exposure and outcome variables. Furthermore, to ascertain the presence of horizontal pleiotropy in MR analysis, the MR-Egger intercept test was conducted. If the intercept term in the MR-Egger analysis yielded statistically significant results (P < 0.05), it indicated significant horizontal pleiotropy. Finally, the Cochran's Q statistic was used to test for heterogeneity. A statistically significant result in the Cochran's Q statistic test (P < 0.05) indicated heterogeneity in the analysis. These tests and sensitivity analyses were conducted to ensure the robustness and validity of the MR results
6	**Statistical methods: main analysis**		Describe statistical methods and statistics used		

		a) Describe how quantitative variables were handled in the analyses (i.e., scale, units, model)		NA
		b) Describe how genetic variants were handled in the analyses and, if applicable, how their weights were selected	7	1. Genome-Wide Significance: SNPs associated with smoking traits should demonstrate genome-wide significance (P < 5×10^-8), referencing data from the European 1000 Genomes Project. 2. Physical Distance and Linkage Disequilibrium: SNPs should have a physical distance greater than 10,000 kb between them, and the LD threshold between genes should be less than an r^2 value of 0.001. 3. Removal of Palindromic Alleles: Palindromic alleles were excluded. 4. PhenoScanner Database: SNPs associated with potential confounding factors related to CTS were removed using the PhenoScanner database. 5. F-Statistic Threshold: SNPs with an F-statistic less than 10 were excluded. A low F-statistic suggests the presence of weak instrument bias, which may affect the results. The F-statistic for a single SNP is calculated as F = (beta/se)^2[23].
		c) Describe the MR estimator (e.g. two-stage least squares, Wald ratio) and related statistics. Detail the included covariates and, in case of two-sample MR, whether the same covariate set was used for adjustment in the two samples	7	The primary method employed in this study is the Inverse Variance Weighted (IVW) method, Additionally, MR-Egger regression and Weighted Median methods were utilized as supplementary analyses
		d) Explain how missing data were addressed		NA
		e) If applicable, indicate how multiple testing was addressed		NA
7	**Assessment of assumptions**	Describe any methods or prior knowledge used to assess the assumptions or justify their validity	5	Previous MR analyses have identified obesity and diabetes as risk factors for CTS
8	**Sensitivity analyses and additional analyses**	Describe any sensitivity analyses or additional analyses performed (e.g. comparison of effect estimates from different approaches, independent replication, bias analytic techniques, validation of instruments, simulations)	7	The MR-Presso method was employed to detect outliers. If outliers were identified, they were removed, and the analysis was repeated. Sensitivity analysis using the "leave-one-out" method involved iteratively removing one SNP at a time to assess whether specific variants were driving the association between exposure and outcome variables.<br>Finally, a meta-analysis was conducted on the MR results (IVW) for both the discovery and validation sets.
9	**Software and pre-registration**			

		a)	Name statistical software and package(s), including version and settings used	8	The analyses were conducted using R packages, including TwoSampleMR (0.5.8), ieugwasr (0.2.1), metafor (4.6.0) and MRPRESSO (1.0).
		b)	State whether the study protocol and details were pre-registered (as well as when and where)		NA
	**RESULTS**				
10	**Descriptive data**				
		a)	Report the numbers of individuals at each stage of included studies and reasons for exclusion. Consider use of a flow diagram	8	We excluded SNPs associated with BMI (rs9835772, rs6265, etc.), type 2 diabetes (rs62107261), risk-taking behavior (rs326341, rs12244388, etc.), and trauma (rs3896224).
		b)	Report summary statistics for phenotypic exposure(s), outcome(s), and other relevant variables (e.g. means, SDs, proportions)	8	The final selected SNPs all have an F value greater than 10
		c)	If the data sources include meta-analyses of previous studies, provide the assessments of heterogeneity across these studies	6	There was no significant heterogeneity in the meta-analysis of the above GWAS studies
		d)	For two-sample MR:<br>i. Provide justification of the similarity of the genetic variant-exposure associations between the exposure and outcome samples<br>ii. Provide information on the number of individuals who overlap between the exposure and outcome studies	6	There was no significant sample overlap between GWAS for exposures and outcomes
11	**Main results**				
		a)	Report the associations between genetic variant and exposure, and between genetic variant and outcome, preferably on an interpretable scale		NA
		b)	Report MR estimates of the relationship between exposure and outcome, and the measures of uncertainty from the MR analysis, on an interpretable scale, such as odds ratio or relative risk per SD difference	9	smoking initiation (OR: 1.17, 95% CI: 1.08-1.27, P < 0.001), smoking status (OR: 1.87, 95% CI: 1.56-2.24, P < 0.001), and lifetime smoking (OR: 2.46, 95% CI: 2.03-3.00, P < 0.001).never smoking is a protective factor against CTS. The meta-analysis summary result for this is (OR: 0.55, 95% CI: 0.42-0.71, P < 0.001).
		c)	If relevant, consider translating estimates of relative risk into absolute risk for a meaningful time period		NA
		d)	Consider plots to visualize results (e.g. forest plot, scatterplot of associations between genetic variants and outcome versus between genetic variants and exposure)	9	MR-Egger and Weighted Median results also indicated a positive association between smoking and CTS (Fig2)

12	**Assessment of assumptions**				
		a)	Report the assessment of the validity of the assumptions	9	Sensitivity analysis indicated no significant heterogeneity or pleiotropy in our MR analysis. Leave-one-out analysis demonstrated that individual SNPs did not substantially influence the results, suggesting the robustness of our MR analysis
		b)	Report any additional statistics (e.g., assessments of heterogeneity across genetic variants, such as I^2, Q statistic or E-value)		NA
13	**Sensitivity analyses and additional analyses**				
		a)	Report any sensitivity analyses to assess the robustness of the main results to violations of the assumptions	9	Sensitivity analysis indicated no significant heterogeneity or pleiotropy in our MR analysis. Leave-one-out analysis demonstrated that individual SNPs did not substantially influence the results, suggesting the robustness of our MR analysis
		b)	Report results from other sensitivity analyses or additional analyses	9	Since there was no heterogeneity in the above meta-analyses, a fixed-effects model was used. Sensitivity analysis indicated no significant heterogeneity or pleiotropy in our MR analysis
		c)	Report any assessment of direction of causal relationship (e.g., bidirectional MR)	10	Smoking initiation, smoking status, lifetime smoking, and never smoked are mutually verified, and it is concluded that smoking is a risk factor for CTS.
		d)	When relevant, report and compare with estimates from non-MR analyses	10	However, a meta-analysis in 2022 incorporating 13 cross-sectional studies, 10 case-control studies, and 8 cohort studies, showed only one cross-sectional study demonstrating an association between smoking and CTS. The observed association in cross-sectional studies may be influenced by confounding factors[31]. This inconsistency with our study's results could be attributed to the lower quality of literature included in the meta-analysis.
		e)	Consider additional plots to visualize results (e.g., leave-one-out analyses)	9	Leave-one-out analysis demonstrated that individual SNPs did not substantially influence the results, suggesting the robustness of our MR analysis (Fig3).
	**DISCUSSION**				

14	**Key results**	Summarize key results with reference to study objectives	10	Smoking initiation, smoking status, lifetime smoking, and never smoked are mutually verified, and it is concluded that smoking is a risk factor for CTS.
15	**Limitations**	Discuss limitations of the study, taking into account the validity of the IV assumptions, other sources of potential bias, and imprecision. Discuss both direction and magnitude of any potential bias and any efforts to address them	11	due to constraints of GWAS data, we could not consider gender factors. Furthermore, our study cannot address unobserved pleiotropy, thus results may be subject to some degree of bias. It should be noted that the causal relationship identified in this study is based on genetic evidence, and further clinical trials are needed to validate this relationship.
16	**Interpretation**			
	a)	Meaning: Give a cautious overall interpretation of results in the context of their limitations and in comparison with other studies	11	Therefore, the conclusions of this MR study should be interpreted with caution.
	b)	Mechanism: Discuss underlying biological mechanisms that could drive a potential causal relationship between the investigated exposure and the outcome, and whether the gene-environment equivalence assumption is reasonable. Use causal language carefully, clarifying that IV estimates may provide causal effects only under certain assumptions	10	Smoking is known to reduce blood circulation and induce nerve fibrosis[27, 28]. Nicotine and carbon monoxide, two hazardous substances found in cigarette smoke, cause oxidative stress, endanger arterial walls, and accelerate the accumulation of fat plaques in blood vessels. Additionally, carbon monoxide in smoke decreases the blood's oxygen-carrying capacity[15]. Smoking cessation enhances the function of high-density lipoprotein and cholesterol efflux, thereby reducing the risk of plaque formation
	c)	Clinical relevance: Discuss whether the results have clinical or public policy relevance, and to what extent they inform effect sizes of possible interventions	11	Advising CTS patients to abstain from smoking could be beneficial for their health.
17	**Generalizability**	Discuss the generalizability of the study results (a) to other populations, (b) across other exposure periods/timings, and (c) across other levels of exposure	11	due to constraints of GWAS data, we could not consider gender factors.
	**OTHER INFORMATION**			
18	**Funding**	Describe sources of funding and the role of funders in the present study and, if applicable, sources of funding for the databases and original study or studies on which the present study is based	13	This work was supported by funding from the Natural Science Foundation of Tianjin (No. 23JCYBJC00700).
19	**Data and data sharing**	Provide the data used to perform all analyses or report where and how the data can be accessed, and reference these sources in the article. Provide the statistical code needed to reproduce the results in the article, or report whether the code is publicly accessible and if so, where	12	The GWAS data for Smoking initiation, Smoking status, Never smoked, and CTS (discovery set) can be downloaded from the IEU database (https://gwas.mrcieu.ac.uk/datasets/) with the registration numbers ieu-b-4877, ebi-a-GCST90029014, ukb-d-20116_0, ebi-a-GCST90018813. Lifetime smoking GWAS summary data is available for download at

				https://doi.org/10.5523/bris.10i96zb8gm0j81yz0q6ztei23d. CTS (validation) data in the FinnGen database download: https://storage.googleapis.com/finngen-public-data-r10/summary_stats/finngen_R10_G6_CARPTU.gz.
20	**Conflicts of Interest**	All authors should declare all potential conflicts of interest	13	The authors declare that they have no competing interests.

This checklist is copyrighted by the Equator Network under the Creative Commons Attribution 3.0 Unported (CC BY 3.0) license.

1. Skrivankova VW, Richmond RC, Woolf BAR, Yarmolinsky J, Davies NM, Swanson SA, et al. Strengthening the Reporting of Observational Studies in Epidemiology using Mendelian Randomization (STROBE-MR) Statement. JAMA. 2021;under review.

2. Skrivankova VW, Richmond RC, Woolf BAR, Davies NM, Swanson SA, VanderWeele TJ, et al. Strengthening the Reporting of Observational Studies in Epidemiology using Mendelian Randomisation (STROBE-MR): Explanation and Elaboration. BMJ. 2021;375:n2233.

© 2025 Shi W. et al.
